# Perspectives on systematic capacity building in pharmaceutical regulation for regulators of medical products

**DOI:** 10.3389/fmed.2024.1394562

**Published:** 2024-04-11

**Authors:** Luther Gwaza, Andrew Chemwolo, Mario Musonda, Rutendo Kuwana, Admire Dube

**Affiliations:** ^1^Health Products Policy and Standards Department, Access to Medicines and Health Products Division, World Health Organization, Geneva, Switzerland; ^2^Regulation and Prequalification Department, Access to Medicines and Health Products Division, World Health Organization, Geneva, Switzerland; ^3^School of Pharmacy, University of Western Cape, Cape Town, South Africa

**Keywords:** regulatory system, competency-based education, capacity building, competency framework, medicine regulators, learning programs, public health, human performance improvement

## Abstract

Having a robust, integrated regulatory system is important for ensuring the availability of safe and efficacious medical products of good quality and for protecting public health. However, less than 30% of countries globally have reached the required regulatory maturity level three, with low- and middle-income countries facing challenges in attracting and retaining qualified staff. World Health Organization (WHO) advocates for systematic workforce development, including competency-based education, to address these gaps. We provide perspectives on a systematic approach to capacity building of medicine regulators based on the experience and lessons learnt in developing and piloting the WHO global competency framework for medicine regulators through three scenarios. A systematic approach to capacity building, such as the human performance technology model, can be used to implement the WHO competency framework as part of organizational performance improvement while ensuring that initiatives are well-defined, targeted, and aligned with organizational goals. The competency framework can be used in different contexts, such as improving organization performance for individual regulatory authorities, strengthening regional collaborations, harmonization and reliance on medical products assessment and joint good manufacturing practices inspections of pharmaceutical manufacturers, and developing learning programs for medicine regulators. A competency-based learning approach for regulatory professionals ensures the transfer of learning to the workplace by integrating real-world practices in learning activities and assessments. Further work is required to develop and validate the assessment instruments, apply the competency framework in other contexts, expanding the learning programmes while continuously providing feedback for further refinement of the competency framework and implementation support tools.

## 1 Introduction

Having a robust, integrated regulatory system at maturity level three, as defined by the World Health Organization (WHO), is essential for ensuring the timely availability of safe and efficacious medical products of good quality and protecting public health from substandard and falsified products (1). Adapted from the International Organization for Standardization’s organizational maturity levels, the WHO global benchmarking tool defines level three as a stable, well-functioning and integrated regulatory system performing all key regulatory functions (1, 2). However, less than 30% of countries globally are confirmed to have their regulatory systems functioning at this maturity level or better (2). This low regulatory capacity, mainly in low- and middle-income countries (LIMCs), highlights a major problem: the lack of adequate human resources with appropriate expertise in medicine regulatory bodies.

Many regulators in LMICs struggle to attract and retain qualified and experienced staff due to a variety of reasons, including a limited supply of people with the appropriate academic and scientific background and high staff turnover due to migration (3). The gap in human resource capacity is further exacerbated by a lack of continuous learning opportunities in pharmaceutical and regulatory sciences, which is vital to keep pace with scientific advancements (4). The current status quo and approaches to address the human resource capacity gaps for regulators in LMICs are not sufficient. At best, the effectiveness and transfer of learning to the workplace of most training opportunities available to LMICs are not apparent, and, at worst, they exacerbate the situation (4). In some cases, training programmes are not needs-based or context-specific and remain focused on the acquisition of knowledge without the application of knowledge and skills to the workplace. Consequently, WHO and others advocate for systematic workforce development, including competency-based education (CBE), to address these gaps (5–9).

A competency framework specifies the organizational requirements for knowledge, skills, attitudes, and practices acquired through education, training and experience (5, 10). WHO published a global competency and outcomes framework for universal health coverage (5) to guide the integration and use of global competencies in the design, delivery and assessment of CBE programmes and specific frameworks, such as one for health workers in primary care (11) and another for medicine regulators (12). Here, we provide perspectives on a systematic approach to capacity building of medicine regulators based on the experience and lessons learnt in piloting the WHO framework for medicine regulators in various contexts: individual regulatory authorities, regional collaborations, and specific courses in Africa.

## 2 Global competency framework for regulators of medicine

Within the framework that underpins WHO competency frameworks (5), education outcomes are framed in terms of what the practitioner will do (practice activities) and how they will do it (competencies). The framework describes practice activities as time-limited, trainable, and measurable through the performance of the tasks, while competencies are durable, trainable and measurable through the expression of behaviours. In addition, a task is described as an observable unit of work within a practice activity that draws on knowledge, skills and attitudes. Behaviour is described as an observable conduct towards other people or tasks that expresses competency and is measurable in the performance of tasks. The framework further defines competence as a state of proficiency of a person to perform the practice activities to the set standard, and performance is a function of competence, motivation and opportunity to participate or contribute. With CBE, effective behaviours are learned when situated within the real-world practice and performance of the tasks and not in isolation.

The competency framework for medicine regulators (12) aligns with the functions outlined in the WHO Global Benchmarking Tool (GBT) (1) and expresses the functions as core or role-specific. Core practices are cross-cutting issues specific to the regulation of medical products, including the elements under the “regulatory systems” in the GBT. The role-specific requirements presently cover four roles: reviewers, inspectors, pharmacovigilance and laboratory analysts. Underpinning these regulatory-specific functions are meta-competencies, which are essential for the world of work and, to a large extent, align with the competency domains described in the global competency and outcomes framework for universal health coverage. The framework includes three proficiency levels representing the career or skill acquisition in any domain (13).

## 3 Organizational performance improvement as a driver for workforce development

Grounding capacity-building strategies in theoretical principles and evidence-based practices ensures their effectiveness and impact on organizational performance. Human performance technology (HPT) models, such as the general model from the International Society for Performance Improvement (ISPI) or Gilbert’s Behavior Engineering Model, can be used (14, 15). Adopting a systematic approach to capacity-building is crucial for ensuring that initiatives are well-defined, targeted and aligned with organizational goals. This approach should encompass assessing the organization’s capabilities to identify performance gaps, analyzing the root causes, developing tailored interventions and evaluating the effectiveness of the interventions (14, 16). HPT models can be employed to ensure that the process is aligned with the needs of the organization and is holistic. ISPI model was used in the pilot of the global competency framework for regulators of medicines.

Various tools can be applied to performance assessment in the workplace, such as surveys, reviews of work samples, work diaries, direct observation, case studies, process mapping, literature reviews, focus groups, reviews of critical incidents, and portfolios to ensure the authenticity of the assessments (17–19). Alternatively, simulations and standard examinations could be applied; however, these are more cost-effective when applied across organizations. In the workplace, assessments should be integrated into routine operations, performed at regular intervals for formative assessments and from multiple sources. Summative assessments should include a minimum of two methods to improve the reliability and validity of the reported results and be tailored to the specific roles or areas to be assessed.

A range of appraisals can be applied, for example, self-appraisal, peer or supervisor appraisal and external assessors. Self-appraisal, although prone to bias, is essential for professional development as it allows ownership of professional and skills development and the development of self-efficacy and self-awareness competencies. A peer or supervisor assessment ensures the internal validity of the results. In contrast, external assessment ensures the external validity of the results, notably to support regional work or recognition of acquired competencies beyond the institution. At a minimum, workplace performance assessment should include self and peer or supervisor assessments. Training of staff and appraisers is essential to ensure the correct interpretation of the performance standards and evaluation of the evidence. Factors to consider in the selection of the assessment format should include reliability, cost-effectiveness and feasibility, and validity and impact.

Next, are perspectives from scenarios under which this approach was piloted using the global competency framework for medicine regulators.

### 3.1 Scenario 1: implementing competency framework in individual NRAs

#### 3.1.1 Step 1: adapting the competency framework

This step involves the senior management defining the desired performance, which correlates with the organization’s strategic plans and benchmarking results. In this step, the essential competencies are identified from the global competency framework, which is then tailored to the organization’s specific needs. The output of this step is a competency manual that has been adapted for the national regulatory authority (NRA). If some roles or functions are not defined in the WHO Global Competency Framework for Medicine Regulators, the management can adapt competencies from other competency dictionaries.

#### 3.1.2 Step 2: competency assessment

Measure actual staff performance against the desired competency profile for their roles to identify gaps. Table 1 provides examples of assessment methods that can be used, and these should be tailored to specific roles or job functions. For example, survey and work samples (assessment reports) could be sufficient for reviewers/dossier reviewers, while survey, work samples (inspection reports) and observed audits would suffice for GMP inspectors. Surveys are preferred at the beginning for their ease and cost-effectiveness in the initial phase, but other methods provide a more comprehensive view. Conclusive evidence should be based on at least two or more assessment methods. At a minimum, the assessment should be self-assessment and peer or supervisor assessment.

**Table 1:** Assessment formats, utility considerations, and relevance to specific roles, and learning and performance outcomes.

Examples of assessment formats	Utility considerations					Relevance to roles				Learning objectives			Performance outcomes	
	Reliability	Validity	Costs (develop/administer)	Operational feasibility (workplace/learning settings)	Predictability	Reviewers	Inspectors	PV	Lab analyst	Knowledge	Skills	Attitude	Behaviour	Practice activities
Survey/checklist	Low	Low?	Low/low	High/high	Predictable	Yes	Yes	Yes	Yes	(√)	(√)	(√)	√	√
Work diaries	Low – moderate	Moderate	Low/high	High/low	Less predictable	No	No	Yes	Yes	(√)	(√)	(√)	√	√
Review of work samples	Moderate – high	High	Low/high	High/low	Predictable	Yes	Yes	Yes	Yes	(√)	(√)	(√)	√	√
Direct observation	Moderate – high	High	Low/high	High/low	Less predictable	No	Yes	No	Yes	(√)	(√)	(√)	√	√
Portfolios	Moderate – high	High	Low/high	High/low	Predictable	Yes	Yes	Yes	Yes	(√)	(√)	(√)	√	√
Proficiency testing	High	High	High/high	High/low	Predictable	No	No	No	Yes	(√)	(√)	(√)	√	√
Process mapping	Moderate	Varies	High/high	High/low	Predictable	No	Yes	Yes	Yes	(√)	(√)	(√)	√	√
Literature reviews	Moderate – high	Varies	Low/high	Moderate/high	Less predictable	Yes	Yes	Yes	Yes	√	(√)	(√)	√	√
Reviews of critical incidents	Low	High	Low/high	High/low	Less predictable	No	Yes	Yes	No	(√)	(√)	(√)	√	√
Simulations	High	Moderate	High/high	Low/moderate	Predictable	Yes	Yes	Yes	No	(√)	√	(√)	√	√
Case studies	High	Moderate	High/high	Moderate/high	Predictable	Yes	Yes	Yes	Yes	(√)	√	(√)	(√)	(√)
Essays	High	Moderate	Low/high	Low/high	Predictable	Yes	Yes	Yes	Yes	√	(√)	(√)		
MCQs, short answer	High	Low	High/low	Moderate/high	Predictable	Yes	Yes	Yes	Yes	√				

(√) Inferred, √ explicit. PV, pharmacovigilance.

The assessments should be performed at regular intervals, for example, annual or biannual, or in specific circumstances such as a change in roles or functions for specific individuals, during recruitment or onboarding, or a change of assignments/position descriptions. The data collection could be integrated into routine practice as this is cost-effective. For example, the work sample assessment rubrics could be integrated into the routine peer review system for dossier assessments for marketing authorizations. At regular intervals, the documented feedback can be reviewed to provide a comprehensive picture of the staff’s competence in authentic, real-world situations. Performing data collection for assessment methods, such as observed audits or analytical testing, during routine GMP inspections and quality control testing is more feasible and cost-effective rather than standalone exercises.

#### 3.1.3 Step 3: cause analysis

A root cause analysis of competence gaps should be conducted, considering workplace, work level and performer level factors. The workplace level addresses the broader organizational systems, structures, policies, and practices, while the work level focuses on specific tasks, processes, or activities within the organization. Performer or worker level refers to the individual employees (14). This step is critical to ensure that interventions target the real issues rather than superficial symptoms. Authentic cause analysis requires knowledge of the assessment tools used and the operating environment of the organization. Table 2 can be used as a reference with some potential causes that the staff can consider in their root cause analysis. Other tools, such as “5 whys analysis,” fishbone diagrams, or failure mode and effects analysis, could be useful in this process.

**Table 2:** Sample of a summary of potential causes of the gaps and interventions for assessments and inspection activities.

Category	Potential causes	Category	Interventions – @ workplace	Interventions – @ work level	Interventions – @ worker level
Report org and clarity	Absence of clear and relevant performance descriptions/expectations.	Motivation and expectations	Establish appropriate performance targets and work specifications/standards.	Allocate tasks based on the skill level and complexity of the task.	
	Inadequate or incomplete information (instructions, SOPs, procedures, or directions).	Data or information	Update the relevant organizational policies or procedures.	Provide current information (instructions, SOPs, or directions).	Awareness and training on the SOPs and procedures.
	Insufficient time to get the job done using best practices.	Motivation and expectations	Job analysis and setting realistic performance goals with respect to quality and quantity of outputs.	Standardize the duration of assessments/inspections (best practice) and appropriate workload management.	Learning opportunities to enhance proficiency and skill acquisitions.
	Lack of best practices in assessments and inspection processes.	Environment support, resources, and tools.	Update the relevant organizational policies or procedures.	Revise and standardize assessment/inspection templates as well as the SOPs to adopt best practices.	Awareness and training on the SOPs and procedures.
	Inadequate peer review and QA mechanisms to assure the quality of the work outputs.	Environment support, resources, and tools.	Update the relevant organizational policies or procedures.	Peer review and QA mechanisms to assure the quality of the work outputs.	
	Lack of good models of behaviour.	Motivation and expectations	Implement staff exchanges/rotations [internal/external].		Seek coaching/mentoring.
Quality of writing	Absence of clear and relevant performance descriptions/expectations.	Motivation and expectations	Establish appropriate performance targets and work specifications/standards.	Allocate tasks based on the skill level and complexity of the task.	
	Lack of good models of behaviour.	Motivation and expectations	Implement staff exchanges/rotations [internal/external].		Seek coaching/mentoring.
	Lack of structured, constructive, educational, and timely feedback mechanisms on performance.	Feedback		Establish structured, constructive, educational, and timely feedback mechanisms in the review and work procedures.	Provide routine, constructive and timely feedback to the performer.
Completeness of report	Variation in assessment/inspection templates and formats used at the country level compared to the standard applied in the joint activities and used in the appraisal process.	Data or information	Update the relevant organizational policies or procedures	Revise and standardize assessment templates as well as the SOPs.	Awareness and training on the SOPs and procedures
				Provide job aids/checklists (scaffolding), especially for less experienced staff.	
	Lack of clearly defined and awareness of the consequences of actions, rewards, or incentives on the achievement of required results.	Consequences, incentives, rewards	Establish clear expectations with consequences, incentives, and rewards for achieving/failing to achieve the desired and agreed results.	Monitor performance and results against established work specifications.	
	Insufficient time to get the job done using best practices.	Motivation and expectations	Job analysis and setting realistic performance goals with respect to quality and quantity of outputs.	Standardize the duration of assessments (best practice) and appropriate workload management.	Learning opportunities to enhance proficiency and skill acquisitions. More skilled workers are efficient.
Scientific rigor	Lack of relevant knowledge and skills on topical issues.	Skills and knowledge		Provide job aids/checklists (scaffolding), especially for less experienced staff.	Learning opportunities to enhance knowledge and skill acquisition. *[self-study, training, advanced studies]*
	Lack of best practices in assessments and inspection processes.	Environment support, resources, and tools.	Update the relevant organizational policies or procedures.	Revise and standardize assessment/inspection templates as well as the SOPs to adopt best practices.	Awareness and training on the SOPs and procedures.
	Use of outdated guidelines and requirements	Environment support, resources, and tools.	Update guidelines and regulations		Training on the relevant guidelines
	Lack of access to current and relevant information (applicable guidelines, reference texts, pharmacopoeias)	Environment support, resources, and tools.	Provide access to relevant materials and resources (guidelines, texts, and pharmacopoeias, e.g., online access).		Training on the relevant guidelines
	Inadequate peer review and QA mechanisms to assure the quality of the work outputs.	Environment support, resources, and tools.	Update the relevant organizational policies or procedures	Peer review and QA mechanisms to assure the quality of the work outputs.	
	Lack of structured, constructive, educational, and timely feedback mechanisms on performance.	Feedback		Establish structured, constructive, educational, and timely feedback mechanisms in the review and work procedures.	Seek feedback.
			Provide coaching/mentoring.	Coaching/mentoring platforms or opportunities	
Summary of critical issues, opinions, and observations.	Lack of relevant knowledge and skills on topical issues.	Skills and knowledge	Implement staff exchanges/rotations [internal/external].	Provide job aids/checklists (scaffolding), especially for less experienced staff.	
	Use of outdated guidelines and requirements	Environment support, resources, and tools.	Update guidelines and regulations		Training on the relevant guidelines
	Insufficient time allocated to review the products using best practices.	Motivation and Expectations	Job analysis and setting realistic performance goals with respect to quality and quantity of outputs.	Standardize the duration of assessments (best practice) and appropriate workload management.	Learning opportunities to enhance proficiency and skill acquisitions. More skilled workers are efficient.
	Inadequate or incomplete information (instructions, SOPs, procedures, or directions).	Data or information		Provide current information (instructions, SOPs, procedures, or directions).	Awareness and training on the SOPs and procedures
	Inadequate peer review and QA mechanisms to assure the quality of the work outputs.	Environment support, resources, and tools.	Update the relevant organizational policies or procedures	Peer review and QA mechanisms to assure the quality of the work outputs.	Coaching/mentoring
	If incentives are available, they may not ensure a balance of positive and negative incentives in favour of excellent performance, hidden incentives supporting poor performance, and punishment for performing well.	Consequences, incentives, rewards	Establish clear expectations with consequences, incentives, and rewards for achieving/failing to achieve the desired and agreed results.	Monitor performance and results against established work specifications.		
	Lack of structured, constructive, educational, and timely feedback mechanisms on performance.	Feedback		Establish structured, constructive, educational, and timely feedback mechanisms in the review and work procedures.	Seek feedback
Compliance with scientific and regulatory requirements	Lack of relevant knowledge and skills on scientific and regulatory requirements.	Skills and knowledge		Provide job aids/checklists (scaffolding), especially for less experienced staff.	Learning opportunities to enhance knowledge and skill acquisition. *[self-study, training, advanced studies]*
	Use of outdated guidelines and requirements	Environment support, resources, and tools.	Update guidelines and regulations		Training on the relevant guidelines
	Lack of access to current and relevant information (applicable guidelines, reference texts, pharmacopeia’s)	Environment support, resources, and tools.	Provide access to relevant materials and resources (guidelines, texts, and pharmacopoeia, e.g., online access).		Training on the relevant guidelines
	Inadequate or incomplete information (instructions, SOPs, procedures, or directions).	Data or information		Provide current information (instructions, SOPs, procedures, or directions).	Awareness and training on the SOPs and procedures
	Inadequate peer review and QA mechanisms to assure the quality of the work outputs.	Environment support, resources, and tools.	Update the relevant organizational policies or procedures	Peer review and QA mechanisms to assure the quality of the work outputs.	Coaching/mentoring
	Lack of structured, constructive, educational, and timely feedback mechanisms on performance.	Feedback		Establish structured, constructive, educational, and timely feedback mechanisms in the review and work procedures.	Seek feedback.
Observations and questions	Lack of relevant knowledge and skills on scientific and regulatory requirements.	Skills and knowledge			Learning opportunities to enhance knowledge and skill acquisition. *[self-study, training, advanced studies]*
	Inadequate or incomplete information (instructions, SOPs, procedures, or directions).	Data or information	Update the relevant organizational policies or procedures	Provide current information (instructions, SOPs, procedures, or directions).	Awareness and training on the SOPs and procedures
	Inadequate peer review and QA mechanisms to assure the quality of the work outputs.	Environment support, resources, and tools.	Update the relevant organizational policies or procedures	Peer review and QA mechanisms to assure the quality of the work outputs.	Coaching/mentoring
	Lack of structured, constructive, educational, and timely feedback mechanisms on performance.	Feedback		Establish structured, constructive, educational, and timely feedback mechanisms in the review and work procedures.	Provide routine, constructive and timely feedback to the performer.
	Lack of good models of behaviour.	Expectations	Implement staff exchanges/rotations [internal/external].		

QA, quality assurance; SOP, standard operating procedures.

#### 3.1.4 Step 4: intervention selection and design

Interventions should be developed based on the root causes identified considering the same three levels: workplace, work level and performer levels (Table 2). These could range from job redesign and review of standard operating procedures to training improvements. Individual development plans should be updated to reflect the non-instruction interventions and the instruction interventions.

#### 3.1.5 Step 5: implementation and evaluation

The interventions should be implemented with continuous monitoring and evaluation to measure effectiveness and make necessary adjustments. Evaluation should be integrated at each step to ensure continued alignment of the outputs or results with the organization’s strategic plans and results from prior steps. For example, the root causes identified in step 3 should be evaluated in the context of the benchmarking results and general knowledge of the organization, including its strengths and weaknesses.

The global competency framework is flexible and should be customized to align with the specific needs and structure of the NRA and should reflect not only the organization’s priorities and the locally regulated environment but also be realistic based on the stage of maturity of the organization. For instance, the global competency framework categorizes competencies into three distinct proficiency levels, demonstrating a nuanced approach to competency classification. However, this model might not fit all organizational contexts. On the one hand, some NRAs prefer a more simplified, flat structure distinguishing primarily between regulatory specialist roles and managerial roles, emphasizing a clear demarcation between technical or scientific work and oversight responsibilities. On the other hand, some NRAs have hierarchical levels within the regulatory roles, such as junior, senior, principal, and chief, to better reflect the depth of experience and expertise required at each stage. This variety underscores the importance of adapting the competency framework to support the unique objectives and operational dynamics of each NRA, ensuring that they effectively enhance capability development and performance management.

Staff involvement and training are essential to mitigate bias and ensure relevance, buy-in, and consistent application of assessment tools, enhancing the overall effectiveness of the implementation. Digital tools for data collection and analysis during the assessment phase should be integrated to streamline the process and enhance accuracy.

### 3.2 Scenario 2: implementing competency framework in a regional context/collaboration in medicine assessments and GMP inspections

Implementing a global competency framework in a regional collaborative context follows the same process as individual NRAs but with some adaptations and variations. WHO and others advocate and support regional collaborations among regulators to enhance regulatory capacity by leveraging the expertise within the group and removing duplication of efforts. However, the inherent organizational differences compound the existing individual differences in regional activities where consensus on assessments or inspections is imperative.

Heads of departments or representatives from individual NRAs collectively define the desired performance for the regional collaboration (step 1), correlating with regional collaboration goals and results of individual benchmarking of the NRAs. This step involves identifying essential competencies from the WHO Global Competency Framework for Medicine Regulators for specific roles in the regional collaboration tailored to the needs of the region. The output is standardized competency role profiles; for example, in the context of regional collaboration on medical products assessment, this would be GMP inspectors and dossier reviewers (such as quality, clinical, pharmacokinetic/bioequivalence reviewers).

After that, steps 2–5, as described under scenario 1, apply. Apart from the supervisor/manager appraisals, peer review within a technical function at the NRA level could be beneficial (internal validation) before the regional validation (external assessments).

Competency role profiles in a regional context should be standardized, notwithstanding structures or practices in individual NRAs.

### 3.3 Scenario 3: short course on bioequivalence reviews

Table 3 shows an extract from a syllabus for a learning program using the CBE approach aimed at equipping bioequivalence reviewers at NRAs with the essential competencies to evaluate bioequivalence data for the registration of multisource medicines. This was piloted in a series of annual bioequivalence training organized by the Medicines Control Authority of Zimbabwe between 2019 and 2021 for medicine reviewers working for national medicines regulatory authorities in African countries. The program progresses through modules addressing specific tasks, competencies and learning outcomes, integrated with practical activities and assessments. This ensures learners not only grasp theoretical concepts but also apply them in real-world scenarios. The terminal objective is to prepare participants comprehensively for assessing bioequivalence studies for oral immediate-release generic products. This entails equipping them with a deep understanding of the scientific, regulatory, and ethical considerations involved, as well as the ability to analyze data critically, make informed decisions, and effectively communicate findings. Achieving this goal signifies readiness to contribute to bioequivalence assessment, ensuring safe and timely access to generic medicines. The program, which includes practical exercises that simulate/use real-world scenarios/data, discussions that enhance critical thinking, and assessments that validate the mastery of competencies, aligns the activities with competencies and outcomes to guarantee a practical, impactful learning experience, culminating in participants’ capability to perform detailed bioequivalence evaluations in line with global standards.

**Table 3:** An extract from the syllabus for a competence-based learning program for the review of bioequivalence data.

Module	Topics covered	Learning outcomes	Learning activities	Assessment
Module 1: introduction to Bioequivalence	Basic concepts of bioequivalence, importance in generic drug approval, regulatory frameworks (WHO, ICH guidelines).	*Recognize and articulate* the scientific and regulatory basis for demonstrating interchangeability.	Lectures, readings.	Quiz on bioequivalence principles.
Module 2: ethical considerations and good clinical practice (GCP)	Ethics in clinical trials, overview of GCP guidelines, ethical oversight mechanisms.	*Explain* the key principles of ethics and GCP in bioequivalence.	An interactive workshop on ethical dilemmas, GCP guidelines discussion.	Written reflection on the ethics case study.
Module 3: study design and conduct	Bioequivalence study designs, dosing regimens, sample size determination, statistical methods.	*Accurately* evaluate study design and identify deficiencies.	Group discussions on study design case studies, lectures on BE principles, clinical study designs	A written critique of a sample study design.
Module 4: study products	Selection of comparator products, understanding test and comparator product characteristics, and assessing product quality.	*Choose* an appropriate comparator product *and conclude* on the acceptability of study products for bioequivalence demonstration.	Role-play exercises to choose appropriate comparator products based on WHO guidelines, analysis of test and comparator product data	Written assignment evaluating the selection and data of test and comparator products for a bioequivalence study.
Module 5: pharmacokinetics and biopharmaceutics in bioequivalence	Basics of pharmacokinetics (PK) and biopharmaceutics, PK parameters, modelling.	*Explain* key principles of biopharmaceutics and pharmacokinetics in bioequivalence.	Interactive PK simulation exercises, biopharmaceutics role-play exercises.	Pharmacokinetics problem-solving exercise. Interpretation and report of pharmacokinetic data
Module 6: bioanalytical method validation	Principles of bioanalysis, method validation criteria (specificity, sensitivity, accuracy, reproducibility).	*Assess* the appropriateness of bioanalytical method validation.	Practical lab sessions, seminar on ICH guidelines for bioanalysis.	Evaluation of a bioanalytical method validation report.
Module 7: assessing Bioanalytical and Pharmacokinetic Data	Reviewing bioanalytical results and PK data analysis for bioequivalence assessment.	*Evaluate* bioanalytical results and *analyze* pharmacokinetic data.	Case study analysis, group projects on PK data interpretation.	Group presentation on bioanalytical and PK data assessment.
Module 8: statistical methods for bioequivalence	Statistical concepts in bioequivalence, analysis techniques, interpreting results.	*Ensure* appropriate statistical analysis of bioequivalence data.	Lecture on statistical methods in bioequivalence, workshop on statistical software, discussions on statistical challenges.	Analysis of statistical data from a bioequivalence study.
Module 9: writing scientific assessment reports	Structure and content of assessment reports, documenting findings, and regulatory documentation.	Write scientific assessment reports that integrate information, highlight key findings and adhere to regulatory guidelines.	BE assessment sessions (full independent BE study review), peer review sessions.	Preparation of a scientific assessment report on a given bioequivalence study.
Module 10: decision making and communication	Making recommendations based on data, communicating findings to stakeholders, and regulatory processes	*Make informed* decisions or recommendations on data acceptability based on evidence and regulatory criteria and *communicate* findings and conclusions effectively (clear, concise and professional writing).	Decision-making scenarios and mock feedback sessions with simulated applicants.	Decision-making exercise based on comprehensive data review, drafting communication to the applicant on observations, deficiencies, and requests for additional information.

Target audience	Reviewers in national medicines regulatory authorities involved in the review of data for marketing authorizations.
Terminal objective	Completed the assessment and reporting on the acceptability of bioequivalence data submitted for registration of multisource (generic) medicines. This includes demonstrating the ability to: Recognize and articulate the scientific and regulatory basis for the demonstration of interchangeability between the generic and comparator products in line with WHO requirements and relevant ICH or equivalent guidelines. Apply key principles of ethics, Good Clinical Practice (GCP), biopharmaceutics, pharmacokinetics, statistics, and bioanalysis in the evaluation of bioequivalence studies. Critically analyze and conclude on the acceptability of available data to demonstrate bioequivalence based on the application of relevant requirements in applicable regulatory guidelines. Effectively communicate findings and recommendations, both in written and oral formats, to stakeholders, ensuring clarity, accuracy, and alignment with regulatory expectations.
Relevant competencies	*Data analysis and interpretation, objectivity in evaluation, evaluation (GCP and study conduct, method validation and bioanalysis of study samples, PK and statistical data), critical thinking, written communication, applying regulatory standards, decision-making, professional attitude towards ethical standards and scientific integrity*
Course delivery model	Virtual or face-to-face with a combination of lectures, workshops/tutorials, online discussion forums, guided assessment practice sessions, and discussions facilitated by tutors and faculty/experts.
Evaluation Method	Continuous assessment through quizzes, written assignments, and a capstone project to evaluate comprehensive understanding and application with real-world bioequivalence data *[appropriate confidential undertakings and declarations of interests for all faculty and participants should be ensured, where necessary permission should be sought from the applicants.]*
Final assessment	Completion of a capstone project involving the full assessment of a bioequivalence study submission, including the preparation of a comprehensive assessment report summarizing findings and making a final recommendation regarding the acceptability of the bioequivalence data. The assessment criteria should include: Quality of analysis: accuracy and depth in assessing the study design, conduct, bioanalytical method validation, pharmacokinetic analysis, statistical analysis, and overall data integrity. Regulatory alignment: the ability to align assessments with current WHO and ICH guidelines, demonstrating an understanding of regulatory requirements and expectations. Communication: the effectiveness of written reports and oral presentations in conveying findings, justifications for conclusions drawn, and recommendations for action. Critical thinking and decision making: demonstrated ability to make informed decisions based on a comprehensive review of all study materials, data, and regulatory guidelines.
Portfolio	Follow up peer-review feedback on the portfolio of completed bioequivalence assessments (three – to six completed BE study reviews).

## 4 Conclusion

Effective capacity building for medicine regulators is essential to protect and promote public health by ensuring the quality, safety and efficacy of medical products available to the public. The capacity building of medicine regulators requires a systematic and comprehensive approach, integrating the WHO Global Competency Framework for Medicine Regulators and, for example, the HPT model. This integration ensures that capacity-building efforts are not limited to training but extend to address various factors influencing organizational performance, such as organization systems, policies and procedures, the use of best practices in the NRAs, and other concepts that are otherwise not sufficiently addressed in traditional approaches, such as the need to improve the self-awareness and self-efficacy concepts of the regulatory staff.

The three scenarios have been presented and demonstrate how the competency framework can be used in different contexts, such as supporting organizational performance improvement, strengthening regional harmonization and collaborations in medical products regulation, and developing learning programmes on regulatory topics to enhance the professional development of regulators. The piloting emphasizes the importance of adapting the competency framework to the specific needs and structure of each NRA and the stage of maturity of the organization.

The perspectives are informed by the limited scope covered in the pilot programme, and there is a need to apply the competency framework in other settings contexts, expand to other regulatory functions/areas and develop and validate the assessment tools. In this context, partnerships and collaboration between the learning context (academic and training institutions) and the performance context (regulators and industry) in designing and delivering learning for regulators are encouraged. Therefore, careful implementation of competency assessments is essential, taking into account the validity, reliability, impact, cost-effectiveness and feasibility of the assessment methods. The selection of assessment methods should be tailored to the roles or functions to be assessed.

## Data availability statement

The original contributions presented in the study are included in the article/supplementary material, further inquiries can be directed to the corresponding author.

## Author contributions

LG: Conceptualization, Formal analysis, Methodology, Project administration, Writing – original draft, Writing – review & editing. AC: Conceptualization, Methodology, Writing – review & editing. MM: Conceptualization, Methodology, Writing – review & editing. RK: Conceptualization, Methodology, Writing – review & editing. AD: Conceptualization, Methodology, Writing – original draft, Writing – review & editing.
